# Supplementary material for: End Sequence Analysis Toolkit (ESAT) expands the extractable information from single-cell RNA-seq data
Source: Genome Res. 2016 Oct;26(10):1397–410. doi: 10.1101/gr.207902.116 (PMC5052061; doi:10.1101/gr.207902.116)
Supplement: Supplemental Material [file supp_gr.207902.116_Supp_Materials.tar.gz › ESAT-esat_v0.1/doc/html/namespacebroad_1_1core_1_1annotation.html]

ESAT: Package broad.core.annotation


ESAT


- Main Page
- Related Pages
- Packages
- Classes
- Files

- Packages

All Classes Namespaces Files Functions Variables Enumerator Pages

- broad
- core
- annotation

Classes

Package broad.core.annotation

|  |  |
| --- | --- |
| Classes | |
| interface | AnnotationFactory< T extends LightweightGenomicAnnotation > |
|  | |
| class | AnnotationFactoryFactory |
|  | |
| interface | AnnotationHandler |
|  | |
| class | AnnotationReader< T extends GenomicAnnotation > |
|  | |
| class | AnnotationReaderFactory |
|  | |
| class | AnnotationUtils |
|  | |
| class | BasicAnnotationReader |
|  | |
| class | BasicGenomicAnnotation |
|  | |
| class | BasicGenomicAnnotationReader |
|  | |
| class | BasicLightweightAnnotation |
|  | |
| class | BasicTwoSubjectAnnotation |
|  | |
| class | BED |
|  | |
| class | BEDGraph |
|  | |
| class | BEDGraphReader |
|  | |
| class | BEDReader |
|  | |
| interface | Feature |
|  | |
| interface | GenomicAnnotation |
|  | |
| interface | GenomicAnnotationFilter< T extends LightweightGenomicAnnotation > |
|  | |
| class | GFF |
|  | |
| class | GFFReader |
|  | |
| interface | LightweightGenomicAnnotation |
|  | |
| class | MaximumContiguousSubsequence |
|  | |
| class | RepeatMaskerAnnotation |
|  | |
| class | RepeatMaskerAnnotationReader |
|  | |
| class | ShortBED |
|  | |
| class | ShortBEDReader |
|  | |
| class | **SimpleAnnotationHandler** |
|  | |
| interface | TwoSubjectAnnotation |
|  | |


---

Generated on Fri Aug 15 2014 15:10:49 for ESAT by  

 1.8.7
